# Effect of multi-strain probiotics as an anti-obesity among overweight and obese Saudi adults

**DOI:** 10.1097/MD.0000000000033245

**Published:** 2023-04-21

**Authors:** Samira M. Almalki, Nasser M. Al-Daghri, Mariam Eid Al-Juhani, Hanan A. Alfawaz

**Affiliations:** a Department of Food Science and Nutrition, College of Food Science and Agriculture, King Saud University, Riyadh, Saudi Arabia; b Chair for Biomarkers of Chronic Diseases, Biochemistry Department, College of Science, King Saud University, Riyadh, Saudi Arabia; c Primary Care Clinic, Applied Medical Science College Clinics – King Saud University, Riyadh, Saudi Arabia.

**Keywords:** adult, obesity, overweight, probiotic supplementation, weight control, weight loss, weight management

## Abstract

Obesity is classified as a low-grade chronic and systemic inflammatory disease and results from complicated interactions between genes and environmental factors, which leads to many diseases and affects the quality of life. There are growing interests in the effectiveness of probiotics as a supplementation to reduce obesity through regulating microbiota host metabolism. Probiotics may influence the interplays among gut, brain, adipose, and liver in a way leading to weight. Since limited studies have been conducted on human subjects, more investigation is needed in this field. Therefore, this study sheds light on the investigation of the anti-obesity effect of probiotic supplementation.

**Methods:**

Ninety adult Saudi overweight or obese adult will be enrolled in this clinical trial and randomized to receive daily placebo or probiotics “MCP® BCMC® strains” for 12 weeks in a double-blind study. Biochemical markers will be measured through blood samples analyzed. Measurements and samples will be obtained at baseline and by the end of the study, at 12 weeks of treatment.

**Discussion:**

This study expects that the multi-strain probiotic product will induce beneficial changes in gut microbiota (GM) including reduction in weight, especially the visceral fat, which leads to reduction in systemic inflammatory state associated with fat accumulation.

## 1. Introduction

The incidence of obesity has increased to be a major health concern in the twenty-first century. According to the World Health Organization, 39% of adults ages 18 years and over were overweight in 2016, and 13% were obese.^[1]^ At the local level, the latest rapid changes in the pattern of Saudi diet have led to exacerbating obesity and overweight to reach 28.7%, 30.7%, respectively, from 15 years and older.^[2]^ Moreover, according to a cross-sectional study has been conducted among school children aged 6 to 16 years in Riyadh city, the overall prevalence of overweight was 13.4% (14.2% for girls and 12% for boys) while obesity was 18.2% (18% for girls and 18.4% for boys).^[3]^ Adult obesity is a major public health problem in Saudi Arabia, if the observed trends continue raising, the prevalence is expected to increase considerably over the next decade from 12% in 1992 to 41% by 2022 in men, and from 21% to 78% in women.^[4]^ Another study has been performed in Jeddah city including 1419 individuals (667 men and 752 women). The spreading of overweight was 35.1 in men and 30.1% in woman, while obesity was 34.8% in men, and 35.6% in women.^[5]^

Confirmed data in humans have shown an association between obesity and the gut microbial community structure.^[6]^ Generally, the human gut has several types of microbes, involving bacteria, archaea, fungi, eukaryotes, and viruses. All these combined organisms compose gut microbiota (GM), which their genes are known as microbiomes.^[7]^ The diversity of the microbiome is affected by many factors such as diet, environment, genetic background, and early microbial exposure. However, in a healthy individual, bacteria are dominant in GM, specifically of the phyla Bacteroidetes and Firmicutes.^[8]^ However, In obese individuals, Turnbaugh et al noticed a significant reduction of GM diversity, also there was a clear lower ratio of Bacteroidetes and a higher ratio of *Actinobacteria* and *Firmicutes* in comparison with lean, this alteration is noticed also in both animal and human studies.

Based on the above, it is evident that a deep understanding of the GM functions helps to find appropriate solutions to control the increasing weight. In animal studies, physical and biochemical parameters, metabolic and inflammatory markers, and alterations in GM diversity revealed beneficial results against obesity whereas the results in humans are still rare. Alteration of the GM via applying natural or supplementation probiotic is considered as a new and promising therapeutic intervention treating human obesity. The present clinical trial aims to study the anti-obesity effects of multi-strain probiotic supplementation on overweight and obese adults.

### 1.1. Aim of the study

Study the anti-obesity effect of consuming multi-strain probiotic supplementation on overweight and obese adults.

### 1.2. Objectives

1. Evaluate the anti-obesity effect of multi-Strain probiotic supplementation.
2. Investigate the effect of probiotics on reducing Lipopolysaccharides LPS.
3. Determine the effect of probiotics on liver functions.
4. Study the association between probiotics and food intake.
5. Evaluate the role of gender difference in response to probiotic.

## 2. Methods

The study is a 12-week, a single center, double-blind, placebo-controlled, randomized, trial to be conducted at the occupational clinical of King Saud University Medical City, Riyadh, Saudi Arabia. Recruitment of subjects will be opened for all students and employees who from King Saud University. The study link will be sent to their email, also they can scan the study code from the banners which are in the active areas at the hospital. A total of 90 overweight or first-class obese subjects, males and females will be recruited and divided equally into 2 groups, a probiotics group, and a placebo group. To be included, a participant should be an adult male or females aged between 19 to 40 years with body mass index (BMI) from 25 to 35 kg/m^2^ and relatively stable body weight in the previous 3 months of the trial.

**Figure 1.**
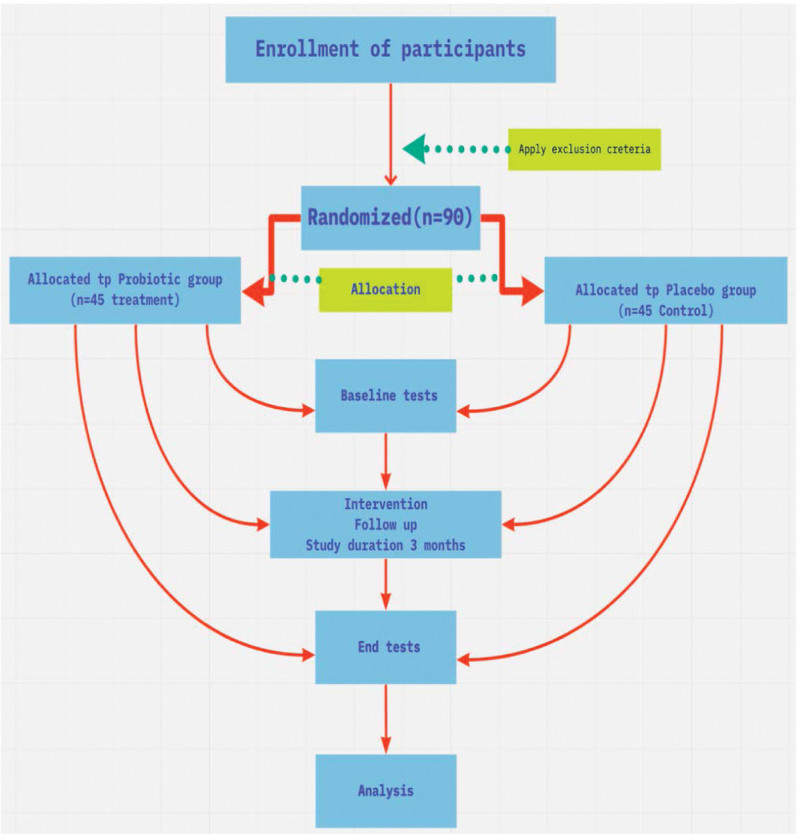
Schematic diagram illustrated the methodology.

Subjects who suffer from diseases and are on treatment, such as immune system diseases or thyroid disorders, pregnant women or who plan to be pregnant, those who had gastrointestinal surgery, hormone replacement therapy, on antibiotics and those who consume probiotic or prebiotic supplements regularly will be excluded. Figure 1 shows the schematic diagram of the study.

The study has been approved by the Institutional Review Board of the College of Medicine in King Saud University (IRB E-20-5503). The study has also been registered in the Saudi Food and Drug Administration registry (SCTR number 21070702) as well as the US clinical trials registry (ClinicalTrials.gov Identifier: NCT05667038).

### 2.1. Intervention

The study is a 12-week, double-blind, randomized, placebo-controlled clinical trial, 90 overweight or first-class obese subjects will be divided equally into 2 groups, a probiotics group, and a placebo group. A hypocaloric diet will be applied for both groups. Interventions will be performed in the first week and then continue to the twelfth week, in which all subjects will receive 3 boxes of either probiotics or placebo, all boxes are identical in color, weight, and shape. All subjects will be instructed to consume 2 sachets per day, first sachet is before the first meal by 10 minutes, and the other one before the last meal by ten minutes. For probiotics sachet, it contains granular powder with 6 microorganism strains (30 × 10^9^ CFU/gram): *Lactobacillus acidophilus* BCMC^®^12130*, Lactobacillus casei* subsp BCMC^®^12313*, Lactobacillus lactis* BCMC^®^12451*, Bifidobacterium bifidum* BCMC^®^02290*, Bifidobacterium infantis* BCMC^®^02129, and *Bifidobacterium longum* BCMC^®^02120. Subjects in the placebo group will receive the placebo consisting of only the carrier of the probiotic product, that is maize starch and maltodextrins.

### 2.2. Outcome measures

Medical history, including physical examination and vital signs, will be recorded before inclusion. Assessment of food frequency consumption for most probiotic and prebiotic food in Saudi culture by using a Food Frequency Questionnaire designed especially for this study. 24-hour dietary recall, all subjects will be asked to record 3 days for their daily intake of food before the intervention, and 3 days by the end of the intervention, in which 2 weekdays and 1 weekend. The first day will be done with a researcher in the clinic, then the other 2 days will be collected by phone call. Anthropometric measurements, the primary outcome of the present study is the relative change in waist circumference (WC) measurement from baseline to end-of-treatment. Other obesity-related outcomes included body weight, WC, and hip circumference. The anthropometrics included:

1. Height will be measured using stadiometer to the nearest 0.5 cm. Subjects should remove shoes, any things cover their head, any cloths that may make it difficult to stand flat against the wall. Standing should be straight, on feet flat on the floor with keeping head, shoulders, and buttocks are touching the wall. The line of sight and chin should be parallel to the floor.^[9]^
2. Body weight will be recorded before breakfast, using a calibrated column scale to the nearest 0.1 kg
3. WC will be measured at the approximate midpoint between the lower margin of the last palpable rib and the top of the iliac crest to the nearest 0.5 cm. To get an accurate waist size, the WC should be taken in a normal respiration situation, and after overnight fasting to reduce stomach contents which may affect the measurement.
4. Hip circumference will be taken around the widest portion of the buttocks, with the tape parallel to the floor. Measuring waist and hip will be obtained by using 150 cm anthropometric measuring stretch-resistant tape ended with steel Hook. The tape should be held snugly around the body, but not in the constricted way. The subject should stand with closed feet, opened arms, with evenly distributing body weight, and should wear little clothing. Also, the subject should be relaxed to avoid any changing in the tension of the abdominal wall. To insure obtain right measuring, measurement should be repeated twice; if the difference is only 1 cm, the average should be counted. If the difference is more than 1 cm, the measurement should be repeated.^[9]^

Physical activity will be assessed at the beginning and the end of the study using the International Physical Activity Questionnaire (short form, last 7 days, self-administered format). Table 1 shows the tasks to be accomplished for each participant visit.

**Table 1:** Visits.

	Pre-visit	First visit	Follow up	Second visit
Pre- screening (through WhatsApp contact)	✔			
Screening	✔	✔		
Inclusion	✔	✔		
Patient attends the occupational clinic		✔		✔
Check eligibility		✔		
Obtain informed consent		✔		
Taking product		✔		
Obtain dietary intake and calculate calories		✔		
Physical activity assessment		✔		✔
Blood sampling		✔		✔
Assessment of applying diet and taking product (through WhatsApp contact or phone call)			✔	
Assessment of compliance and adverse events (through WhatsApp contact or phone call)			✔	
Return remained product				✔

This table shows the tasks to be accomplished for each participant visit.

### 2.3. Blinding

The identity of probiotic and control treatments will not be known to investigators, research staff, or subjects. The following study procedures will be in place to ensure double-blind administration of study treatments:

• Access to the randomization code will be strictly controlled.
• Packaging and labeling of test and control treatments were coded, but shape, color, smell, and weight will be identical.
• Due to necessity of evaluate the activity of treatment product, both packages (with different letters) will be sent to microbiology lab to determine the activity of strains, the result will be blind without mention to any letter.

Unblinding will be done on completion of the clinical study. During the study, the blind may be broken only in emergencies when knowledge of the subject treatment group is necessary for further subject management. In this case, the investigator should discuss the emergency with the Medical Monitor prior to unblinding. The principal investigator (PI) has a direct number-mobile to contact with the company personnel in charge of blinding. The product will be provided in sachets packed and coded as numbers. All subjects will be allocated in blocks according to their gender, age, and BMI. From those blocks, PI will make a list of pairs, each pair has 2 subjects. This list will be sent later to the collaborative members from inpatient pharmacy, who will allocate subjects (1:1) in 2 different groups. The randomization scheme will be computer generated using Excel sheet. After completing the intervention, a request letter will be sent to the company to unblind the study. Then the company lab will send an unblinding letter to inform us which alphabet is probiotic or placebo.

#### 1.2.3. Implementation.

The roles of members in allocation process:

First: PI:

1. Give a unique number for each subject.
2. Sign subjects in blocks according to their gender, age, and BMI.
3. Make a list of pairs and send it the inpatient pharmacy.

Second: Members from inpatient pharmacy:

1. Allocate subjects (1:1) in 2 different groups.
2. Make the randomization scheme by computer generated via Excel sheet.

### 2.4. Sample size calculation and data analysis

The power calculation was based on the results of Gomes and his colleagues who used the G-Power software (version 3.0.10) to calculate the sample size. The primary outcome in that study is the difference in WC between groups. The power calculation requires 17 subjects in each group (95% power; 5% type I error) to detect a difference in WC.^[10]^ In this study, to avoid any later drop-off, both groups will be set to have 45 subjects. Data will be analyzed using SPSS (version 23 Chicago, IL). Categorical data will be presented as frequencies and percentages. t tests and Mann–Whitney tests will be used to determine significant differences between groups at baseline. Also, a mixed-method analysis of covariance and a chi-square test will be used to determine within and between-group differences in some parameters. Intervention effects will be presented with a 95% confidence interval. A P value < .05 will be considered statistically significant.

## 3. Discussion and conclusion

There is a growing interest in the effectiveness of probiotics for treating chronic diseases. This study will evaluate the effects of multi-strain probiotics as an anti-obesity among overweight and obese Saudi adults. Regulating microbiota is considered a potential therapeutic avenue for obesity through many different mechanisms.

The strains which have been used in this study are expected to beneficial effect on weight loss, however, any other results will still be interesting. Previous clinical trials in Saudi Arabia yielded positive results on the effects of multi-strain probiotics among patients with diabetes, specifically favorable outcomes in terms of improving insulin sensitivity.^[11,12]^ Although anthropometric measures were obtained in those trials, no discernible findings were observed over time. The present study aims to focus on the effects of multi-strain probiotics on obese participants without diabetes. Serum analysis of biochemical parameters will be done routinely as done in previous studies.^[13,14]^ Unexpected results will generate new ideas such as adjusting the concentration of strains or looking for new ones. The outcome of this study will be published and will be submitted to the conferences. All participants will receive their results.

## Acknowledgments

The authors acknowledge the research coordinators who are currently supporting the conduct of this trial.

## Author contributions

**Conceptualization:** Samira M. Almalki.

**Data curation:** Mariam Eid Al-Juhani.

**Investigation:** Samira M. Almalki, Nasser M. Al-Daghri, Hanan A. Alfawaz.

**Methodology:** Samira M. Almalki.

**Resources:** Mariam Eid Al-Juhani.

**Supervision:** Nasser M. Al-Daghri, Hanan A. Alfawaz.

**Writing – original draft:** Samira M. Almalki.

**Writing – review & editing:** Nasser M. Al-Daghri, Mariam Eid Al-Juhani, Hanan A. Alfawaz.
